# Inverse Pneumatic Artificial Muscles for Application in Low‐Cost Ventilators

**DOI:** 10.1002/aisy.202000200

**Published:** 2020-10-30

**Authors:** Seyed M. Mirvakili, Douglas Sim, Robert Langer

**Affiliations:** ^1^ Koch Institute Massachusetts Institute of Technology Cambridge MA 02139 USA; ^2^ Electrical and Computer Engineering Department University of British Columbia Vancouver BC Canada; ^3^ Department of Chemical Engineering Massachusetts Institute of Technology Cambridge MA USA; ^4^ Division of Health Science and Technology Massachusetts Institute of Technology Cambridge MA USA; ^5^ Institute for Medical Engineering and Science Massachusetts Institute of Technology Cambridge MA USA

**Keywords:** artificial respirators, low-cost ventilators, pneumatic artificial muscles, point-of-care technology

## Abstract

The procurement and maintenance cost of high‐end ventilators preclude their stockpiles sufficient for the mass emergency situations. Therefore, there is a significant demand for mechanical ventilators in such situations. Herein, a low‐cost, portable, yet high‐performance design for a volume‐controlled mechanical ventilator is proposed. Pneumatic artificial muscles, such as air cylinders, are used in the inverse mode of operation to achieve mechanical ventilation. With the current design, the two fundamental modes of operation (controlled mode and assisted mode) are demonstrated. Unlike most intensive care unit ventilators, the proposed device does not need a high‐pressure air pipeline to operate. The device is capable of mechanical ventilation for respiration rate ranging from 10 to 30 b min^−1^ with a tidal volume (*VT*) range of 150–1000 mL and the *I:E* ratio of 1:1–1:5. A total cost of less than $400 USD is achieved to make one device. The cost to produce the device in larger volumes can be estimated to be less than $250 USD.

## Graphical abstract

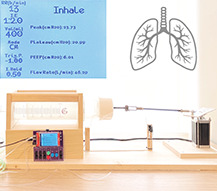

## 1 Introduction

Respiratory dysfunction due to diseases, physical damage to the lungs, or air pollution can be fatal if not treated in time. Artificial ventilation is used to deliver air (e.g., oxygen with air/helium/nitric oxide)—in a pure form or mixed with drugs—to the lungs to assist or replace spontaneous breathing. The two major methods of pulmonary ventilation are *manual* insufflation of the lungs (via mouth‐to‐mouth resuscitation or compressing a bag‐valve‐mask [BVM]) and *mechanical* ventilation of the lungs with electronically or mechanically controlled equipment. The manual method can be sufficient for temporary cardiopulmonary resuscitation (CPR), whereas mechanical ventilation is necessary for prolonged pulmonary ventilation. The state‐of‐the‐art mechanical ventilators address the need for all range of respiratory failures; however, their time‐to‐manufacture, design sophistication, cost, and scalability for deployment in mass emergencies, such as pandemics, make them not a viable solution in many situations.

Delivering air with a compliant bladder, such as a BVM, is considered the simplest yet effective method for pulmonary ventilation. While being sufficient for emergency cases, the BVM manual resuscitators cannot be utilized for prolonged ventilation. Moreover, the operation of BVMs requires training and constant attention of the operator. This tedious and repetitive task can be automated to save the time of a well‐trained medical staff who can then focus on other aspects of resuscitation. MIT's E‐Vent solution addresses this issue by utilizing an electronically controlled mechanical gripper that periodically presses the compliant bladder of the BVM resuscitator.^[^ ^1^ ^]^ As its introduction, a variety of approaches have been proposed and implemented to automate the operation of BVM resuscitators.^[^ ^1^ ^]^ Although automation is achieved, there are several shortcomings associated with interfacing BVM resuscitators with mechanical grippers. Due to the dynamics of deflating the BVM, there can be inconsistencies between breathing cycles. Moreover, it is difficult to monitor the flow rate and volume of air delivered to the lungs due to the compliance of the bag and its geometry. In addition, excessive and prolong compression/decompression of the bag, if not secured, can lead to material fatigue and leakage, which makes it delicate.^[^ ^2^ ^]^

There are two major types of mechanical ventilation: positive pressure ventilation and negative pressure ventilation. In the first type, a positive pressure of air is applied to the lungs to deliver a controllable volume to the respiratory system. In comparison, in negative pressure ventilation, the chest goes under a negative pressure, which expands the lungs and sucks air into the lungs (e.g., iron lung and chest cuirass). Negative pressure ventilators are considered to be noninvasive.

Positive pressure ventilators were first introduced in the early 1950s to treat polio patients with respiratory paralysis.^[^ ^3^ ^]^ They deliver air under a constant volume, pressure, flow rate, or a combination of these parameters. Constant pressure ventilators can be noninvasive or invasive. In noninvasive ventilation, the air is delivered via an interface, such as nasal, oronasal, facial masks, mouthpieces, and helmets. Primary modes for noninvasive ventilation are continuous positive airway pressure (CPAP), auto‐titrating (adjustable) positive airway pressure (APAP), and bilevel positive airway pressure (BiPAP). Unlike the noninvasive ventilation technique, in the invasive technique, the air is delivered with a tube via endotracheal intubation or tracheostomy. Positive pressure ventilators are the most popular type of ventilator. They are used at home (for obstructive sleep apnea), resuscitation in the case of emergency, intensive care units (ICUs), and operation rooms during surgery.

A typical ICU ventilator starts around $2000 and can be as expensive as $50 000.^[^ ^4^ ^]^ In this work, we are demonstrating a fully functional device from readily available components, such as pneumatic artificial muscles (PAM). PAM are among the most widely applied actuators in the industry due to their simple design.^[^ ^5^, ^6^ ^]^ The energy and power density of such artificial muscles do not exceed those of thermal actuators, such as shape memory alloy fibers^[^ ^7^, ^8^ ^]^ and highly oriented semi‐crystalline fibers.^[^ ^9^, ^10^ ^]^ However, in terms of cycle life and performance stability, PAMs are among the most reliable actuators suitable for biomedical devices. Air cylinders, one of the sub‐categories of artificial muscles, convert pneumatic energy to mechanical energy in a cylinder/piston type structure. We are using this operation mechanism in reverse by applying a linear stroke to the actuator to obtain air pressure/volume to perform the mechanical ventilation. For succinctness, we call the device inverse pneumatic artificial muscle (IPAM) ventilator throughout this work.

Because of the advances in manufacturing precision glassware, glass syringes of up to 1000 mL capacity are now commercially available. Traditionally, in chemistry labs, glass syringes are used to smoothly deliver an exact volume of a gas to a system or collect a known volume of gas from a reactor. Unlike plastic syringes, the small coefficient of friction between the plunger and the barrel of the glass syringe offers a smooth profile for flow rate and pressure. In this work, we are utilizing a 1000 mL glass syringe as the pressure source for mechanical ventilation.

In some anesthesia ventilators, smooth and accurate ventilation is achieved using bellows. However, the mechanical work for moving the bellows is provided by the high‐pressure (50 psi) central air pipelines of medical centers. Pneumatic‐powered mechanical ventilators’ reliance on high‐pressure air pipelines makes them less desirable for scenarios where a portable ventilator is needed. Examples of such situations include home care, delivering patients in ambulances, office‐based anesthesia practices, and transferring patients from ICU to other units (e.g., imaging and operation room). Moreover, unlike bellows, glass syringes exhibit low compliance. Therefore, accurate *VT* delivery can be performed.

We believe our proposed solution is reliable for prolonged use and is accurate and low cost (<$400 USD). Moreover, the device is scalable in emergencies and has a rapid manufacturing time. Our design involves eight major components: 1) stepper motor; 2) motor driver; 3) crank‐shaft linkage; 4) 1000 mL glass syringe; 5) power supply; 6) microcontroller; 7) sensor; and 8) interface input/output [i.e., pushbuttons, liquid crystal display (LCD), and switches].

**Figure 1:**
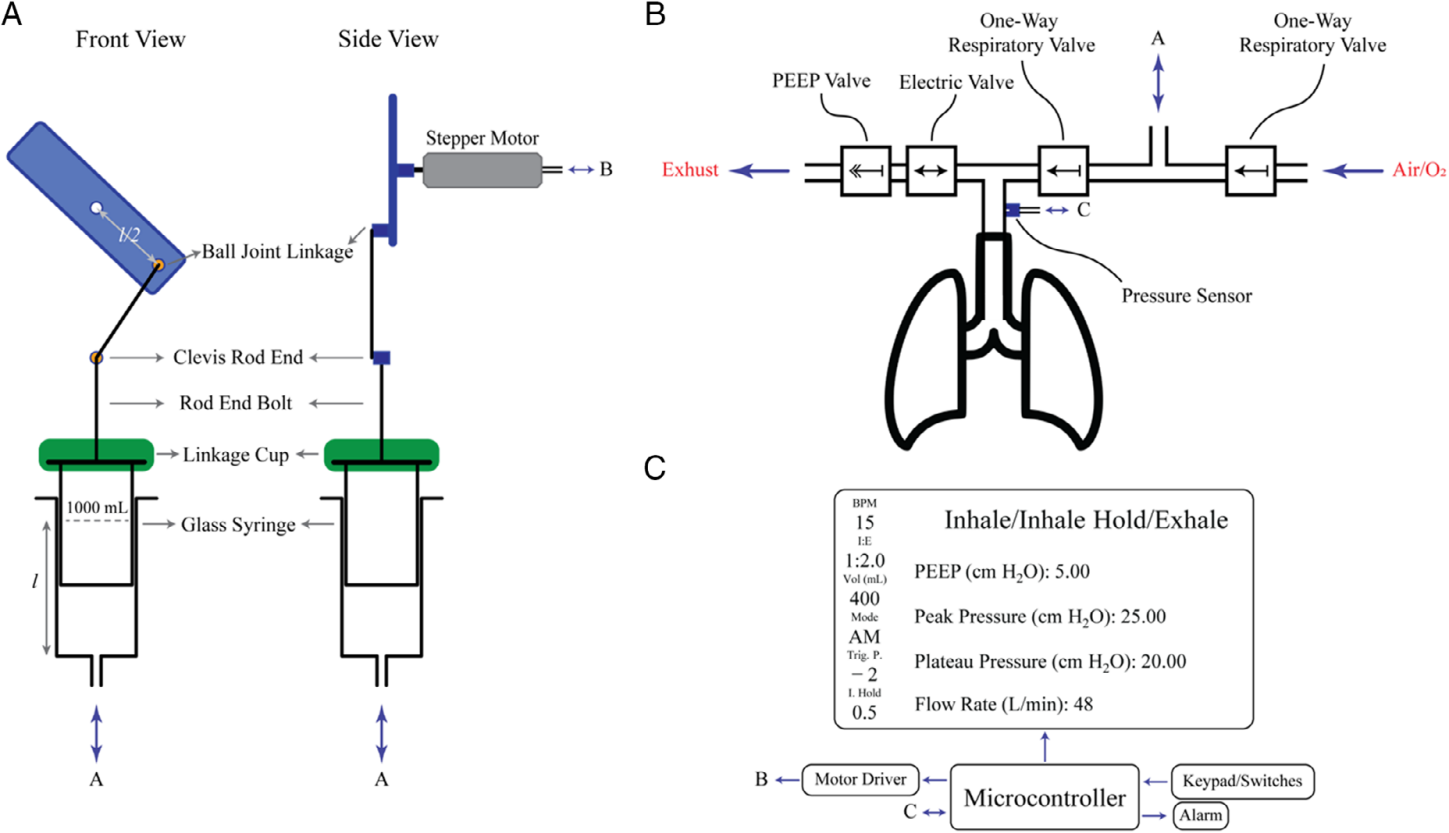
High‐level schematic of the ventilator. A) Illustration of the mechanical system performing the ventilation. B) Schematic of the breathing circuit interfaced with the ventilator and the lungs. In this work, we are employing the breathing circuit of a BVM resuscitator and do not use any electric valves. C) High‐level schematic of the electrical components used in the mechanical ventilators and its user interface.

The mechanical system is responsible for pushing/pulling the plunger of the glass syringe and is digitally controlled by the microcontroller (**Figure** **1**A). The outlet of the glass syringe is connected to the breathing circuit (Figure 1B). The primary role of the breathing circuit is to enable the syringe to take in air from a reservoir bag or the ambient environment when the glass syringe is pulling. The air is then delivered to the lungs when the glass syringe is pushing without escaping from the intake pathway (Figure 1B). In the current design, we are using a mechanical positive end expiration pressure (PEEP) valve, as shown in Figure 1B. It is possible to use a digitally controlled PEEP valve. The user interface lets the operator set parameters, stop/start the device, and monitor the pressure and flow rate on the LCD (Figure 1C).

The plunger of the glass syringe is linked to the crank by two joint linkages. The plunger is first attached to a rod end bolt by a resin (materials and methods). The rod end bolt is connected to a clevis rod end, which can freely rotate by 180°. The clevis rod end is linked to the crank via a threaded rod and a ball joint linkage. Finally, the crank is attached to the stepper motor via a flange‐mount shaft collar. The crank‐shaft linkage converts the rotational motion of the stepper motor into linear motion. The dynamics of the movement can be controlled by applying different pulse functions to the stepper motor (materials and methods).

In this work, we are employing the breathing circuit from a BVM for two reasons. First, the one‐way respiratory valves, the PEEP valve, and the pressure gauge valve are compact and easy to integrate with the IPAM ventilator. Second, in the case of failure, the BVM can be operated manually to resuscitate the patient in place of the device.

The IPAM ventilator can be categorized as an assist‐control ventilator (ACV) device, previously known as the continuous mandatory ventilation (CMV). The device controls the volume and can operate in two modes. The first mode is the *controlled mode* (e.g., CM mode), which is suitable for sedated and paralyzed patients. The second mode is the *assisted mode* (e.g., AM mode) for assisting patients who can breathe spontaneously but need extra volumes of air or higher FiO_2_ (i.e., the fraction of inspired oxygen). Due to the urgent need for ventilators in the current pandemic (COVID‐19), we are only demonstrating the mentioned two modes. More modes of operation can be introduced on the same hardware by adding more program logic. The key parameters that control the function of ventilators include the following.

*VT* is the volume of air displaced between inhalation and exhalation when extra effort is not applied. The gravimetric *VT* is often reported in the units of cc or mL per predicted body weight (PBW; kg). Typical values for patients with acute respiratory distress syndrome (ARDS) are between 4 and 8 mL kg^−1^. The PBW for male and female patients can be estimated from the height of the patients according to the ideal body weight equation described in the following equations.^[^ ^11^ ^]^

(1) $${\text{PWM}}_{\text{male}}(kg)=50+0.91(H-152.4)$$

(2) $${\text{PWM}}_{\text{female}}(kg)=45.5+0.91(H-152.4)$$

where *H* is the height measured in the unit of cm. The range for *VT* is between 70 and 840 mL (Supporting Information).

*Respiratory Rate (RR)*, also known as breath per minute (BPM), is the number of respiration cycles that occur per minute. The typical range for an adult is between 12 and 20 b min^−1^.^[^ ^12^ ^]^ RR of up to 30 b min^−1^ is also reported for COVID‐19 patients.^[^ ^1^, ^13^ ^]^

*Inhalation/exhalation ratio (I:E or IER)* defines the time ratio between the inhale and the exhale cycle. The typical value for a healthy human is 1:2 and is reduced to 1:4 or 1:5 in the presence of obstructive airway disease.^[^ ^14^ ^]^

*End‐inspiratory hold* is defined as the hold time occurring at the end of the inspiration cycle. The end‐inspiratory hold maneuver is performed to eliminate the pressure contribution from the airway resistance and reveal the pressure in the alveoli.^[^ ^15^ ^]^ In the current design, the value is set as the percentage of the inhale time.

*Inhale trigger pressure (ITP or TP)* is only applied in the assisted mode. This represents the slight negative pressure that is developed in the breathing circuit when the body attempts to inhale. By adjusting this parameter, we can ensure the device is synchronized with the spontaneous respiration rate of the patient.

*Positive end‐expiratory pressure (PEEP)* is the gauge pressure in the lungs (alveolar pressure) developed at the end of expiration. The PEEP is typically applied by the ventilator at the end of each breath to reduce the likelihood of the alveoli to collapse. This positive pressure recruits the closed alveoli in the sick lung and improves oxygenation. In this work, we used a mechanical PEEP valve.

Depending on the condition of the patient (e.g., anesthesia, oxygen therapy, obstructive sleep apnea, and severe acute respiratory syndrome), different combinations of parameters are used. For example, for ARDS, which also occurs with COVID‐19, a *VT* of 400–600 mL with an *I*:*E* ratio of 1:2–1:4 with a respiration rate of 12–16 b min^−1^ is used.^[^ ^13^ ^]^

**Figure 2:**
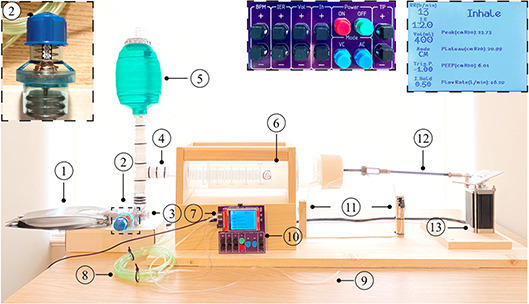
Image of the setup. The components used in this design are: 1) a 1000 mL test lung (compliance: 25 mL mbar^−1^ at a *VT* of 500 mL and a PEEP of 0 mbar. Resistance: 20 mbar L^−1^ s^−1^), 2) PEEP valve—adjustable from 0 to 20 cmH_2_O, 3) mask adaptor of the BVM resuscitator, 4) tubing and tubing adaptors, and 5) resuscitation bag of the BVM with the one‐way intake valve. The reservoir bag for oxygen connects to the top of the BVM bag and is not shown here, 6) 1000 mL BLG glass syringe, 7) differential pressure sensor, 8) tubing connecting one port of the pressure sensor to the mask adaptor, and 9) tubing connected to the free port of the pressure sensor. This tube was only used in the assist mode to simulate inspiration effort to trigger the device. 10) Controller with the keypad and display (*V*1: the *I*_h_ is set in the software), 11) two limit switches, 12) crank‐shaft linkage (materials and methods), and 13) the stepper motor. Insets: top‐right is a zoomed‐in snapshot of the display during ventilation, and the top middle is a zoomed‐in image of the keypad for the *V*2 of the design. In *V*2, the *I*_h_ is set on the keypad. The green button starts the ventilation process, and the stop button stops. BPM, IER, Vol, Mode, and TP are the buttons to set the respiration rate, *I*:*E* ratio, *VT*, mode of operation, and the trigger pressure.

We made a test setup to evaluate the performance of the design. As shown in **Figure** **2**, the setup is made of readily available components. To accelerate the prototyping of the setup, we built the breathing circuit from plastic tubes and tubing adaptors from fast shipping vendors. It is easy to replace the current breathing circuit with low‐cost U.S. Food and Drug Administration approved tubing with close no modification of the device.

For logging data, we used a sampling rate of 50 ms. For low respiration rates, 50 ms sampling is sufficient. However, for higher respiration rates (>25 b min^−1^), to log the data accurately, faster sampling rate is required, which comes at the expense of stressing the microcontroller.

In volume‐controlled mode, the ventilator does not sense any efforts from the patient for breathing. It provides the *VT* at a specific respiration rate and the *I:E* ratio that is configured by the operator. The specifications for the device are listed in **Table** **1**.

**Table 1:** The ventilator device ratings

Parameter	Range
*VT* [mL]	150–1000
Respiration rate [b min^−1^]	6–30
*I:E* ratio	1:1.0–1:5.0
End‐inspiratory hold [%]	0–80
Trigger pressure [cmH_2_O]	−1.0 to −6.0
Positive end exhalation pressure [cmH_2_O]	0–20
Absolute pressure ratings [cmH_2_O]	−163 to 163
Dimensions [mm]	300 × 915 × 250
Weight [kg]	10.4

The pressure is monitored through the whole respiration cycle; however, the *VT* and flow rate are calculated from the position of the stepper motor. Therefore, the volume and the flow rate numbers are representative of the inspiration cycle only, and data for the expiration phase on the plot represent those of the glass syringe. During the expiration cycle, the patient respiratory system is decoupled from the glass syringe, and it is performed independently by the patient via the mechanical recoil of the lungs. To be able to record the patient's volume and flow rate during the exhale cycle, extra peripherals are needed.

**Figure 3:**
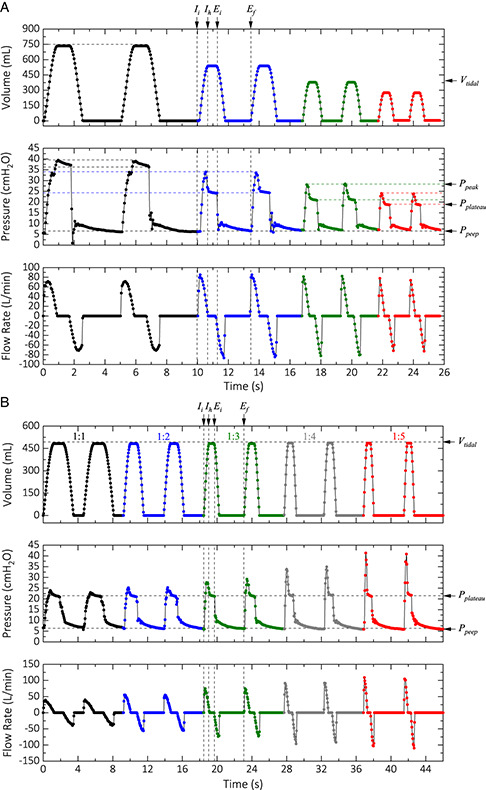
Performance of the ventilator in the controlled mode with a different set of operating parameters. Setting parameters: A) Black: *VT* = 750 mL, *RR* = 12 b min^−1^; blue: *VT* = 550 mL, *RR* = 18 b min^−1^; green: *VT* = 400 mL, *RR* = 24 b min^−1^; red: *VT* = 200 mL, *RR* = 30 b min^−1^. The *I*:*E* ratio and the PEEP were set to 1:2 and 6 cmH_2_O for all the cases. B) The device setting for this case was *VT* = 500 mL, *RR* = 13 b min^−1^ with the *I*:*E* ratio of 1:1 (black), 1:2 (blue), 1:3 (green), 1:4 (gray), and 1:5 (red). The PEEP was set at 6 cmH_2_O for all cases.

To evaluate the performance of the device, we swept the parameters, tested edge cases, and characterized boundary conditions that are used in real‐life scenarios as well. **Figure** **3** shows the volume, pressure, and flow rate for nine test cases (tabulated data in Supporting Information). We performed the end‐inspiratory hold maneuver with a hold time of 50% to obtain the plateau pressure for each case. For experiments in Figure 3A, we kept the *I*:*E* ratio constant at 1:2, but increased the *RR* from 12 to 30 b min^−1^ while decreasing the *VT* from 750 to 300 mL. As we decreased the *VT*, both the peak pressure and plateau pressure decreased as well. This correlation can be explained by the fact that less air is moved to the test lung; therefore, a smaller pressure is developed inside it. For experiments in Figure 3B, we kept the *VT* at 500 mL and the *RR* at 13 b min^−1^ but increased the *I*:*E* ratio from 1:1 to 1:5. In this case, the volume is kept constant. Therefore, the elastic component of the peak pressure (*P*_plateau_ − *PEEP*) is also constant. However, the resistive component of the peak pressure increases (*P*_peak_ − *P*_plateau_). This increase can explain by the fact that the resistive pressure is a function flow rate (Supporting Information). As shown in Figure 3, the cycles are very consistent and repeatable. Any slight discrepancies in the data are most likely due to the under‐sampling of the parameters.

The breathing cycle for the patient consists of three major cycles. The inhale cycle (starting at *I*_i_), the inhale hold cycle (starting at *I*_h_), and the exhale cycle starting at the end of the inhale cycle (*E*_i_) and ends at *E*_f_. The exhale cycle for the glass syringe/motor includes one more cycle: the exhale hold—the flat bottom region on the volume plot in Figure 3. In the exhale hold cycle, the plunger stays in that position (corresponding to the set *VT*) until the next inhale cycle is triggered internally or by the patient's attempt to inhale.

**Figure 4:**
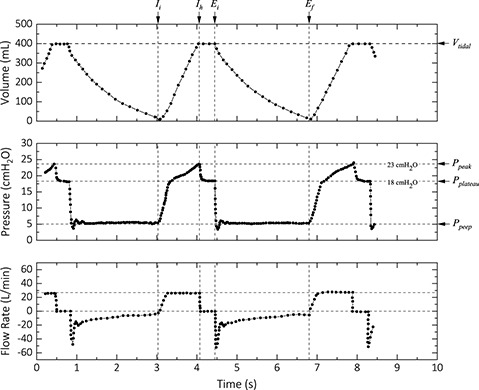
Performance of a Maquet Servo‐I ventilator configured to deliver a *VT* of 400 mL with a PEEP of 5 cmH_2_O and an *I*:*E* ratio of 1:1.7. The plot is regenerated by digitizing an image from the screen of the device under operation for a COVID‐19 patient. Due to the smoothness of the volume and flow rate profiles, a lower spatial sampling rate was used for the digitization.

**Figure 5:**
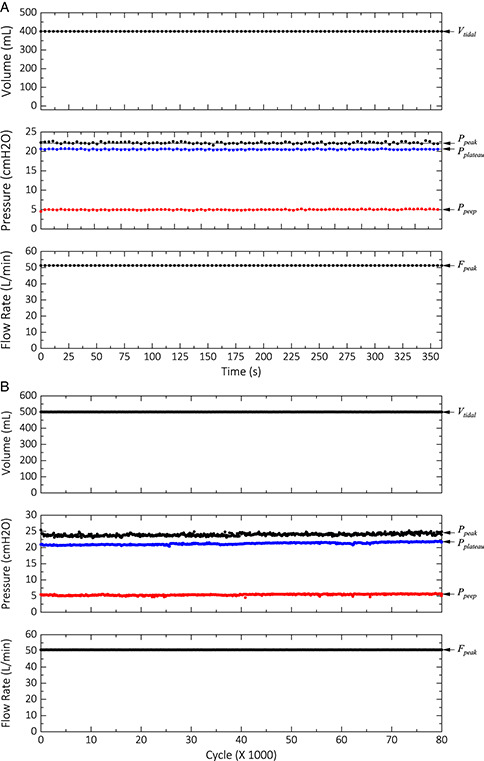
A) Performance of the device for 100 cycles. The test configuration was *VT* = 400 mL, *I*:*E* = 1:1.7, and *RR* = 16 b min^−1^, with an inspiration hold time of 50%. As shown, the timing of the cycles is precise. B) Performance of the device over 80 000 cycles. The test configuration was *VT* = 500 mL, *I*:*E* = 1:2, and *RR* = 12 b min^−1^, with an inspiration hold time of 50%. The slight drift in the PEEP value increased the peak and plateau pressure values as well.

To better evaluate the performance of the IPAM ventilator, we performed ventilation of a test lung under identical device configurations and operating mode used for a COVID‐19 patient with a Maquet Servo‐I ventilator. **Figure** **4** shows the tidal volume, pressure, and flow rate for a configuration of 400 mL delivery at the *I:E* ratio of 1:1.7, a respiration rate of 16 b min^−1^, and a PEEP of 5 cmH_2_O for a COVID‐19 patient. This configuration resulted in a peak pressure of 22 cmH_2_O with a plateau pressure of 20 cmH_2_O with the IPAM ventilator. The slight discrepancies are due to the small differences in the mechanical compliance of the test lung/the breathing circuit that we used and the patient's lung/the breathing circuit that was used with the Maquet Servo‐I ventilator. To test the reproducibility of the IPAM ventilator, we logged the performance parameters for 100 consecutive cycles for this device configuration with an inspiration hold time of 50% (0.7 s). As **Figure** **5**A shows, the performance parameters are very consistent over the entire duration. We measured a mean peak pressure of 22.20 ± 0.22 cmH_2_O, a mean plateau pressure of 20.50 ± 0.10 cmH_2_O, and a mean PEEP of 4.98 ± 0.11 cmH_2_O. We performed a long cycle life test (80 k cycles) under *VT* = 500 mL, *I:E* = 1:2, *RR* = 12 b min^−1^, and inspiration hold = 50%. We measured a mean peak pressure of 23.98 ± 0.37 cmH_2_O, a mean plateau pressure of 21.22 ± 0.36 cmH_2_O, and a mean PEEP of 5.41 ± 0.21 cmH_2_O (Figure 5B). The slight drift in the PEEP value is due to the mechanics of the PEEP valve that we used. This drift manifests itself in the peak and plateau pressure as well.

It is important to note that due to the kinematics of the actuator (Supporting Information), the plunger of the glass syringe does not travel linearly but rather exhibits a sinusoidal profile (supporting information). Therefore, the flow rate has a “sinusoidal” characteristic. However, it is possible to digitally control the stepper motor to obtain a linear profile for the *VT*, similar to that of Figure 4.

The *VT* and flow rate were almost constant, which is due to the fact that in every cycle, the stepper motor's position was recalibrated with the limit switch. Therefore, there was no error, drift, or inconsistencies between successive cycles.

Modern ventilators deliver oxygen (mixed with air) to the lungs and monitor the CO_2_ during expiration with a capnograph. The reservoir bag of the BVM that we used in our design can be filled with oxygen to increase the FiO_2_. Moreover, anesthetic gas agents (e.g., sevoflurane and isoflurane) can be delivered via the medication port of the BVM mask adaptor for patients under anesthesia.

**Figure 6:**
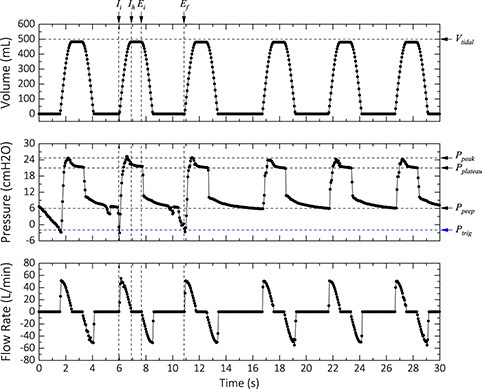
Assisted mode test results. The device was configured to deliver 500 mL at a respiration rate of 12 b min^−1^ with an *I*:*E* ratio of 1:2.

Similar to the controlled mode, in the assisted mode, a constant volume of air is delivered to the lungs. However, in this mode, the inspiratory cycle is triggered by the patient's attempt to inhale. The effort to inhale generates a slight negative pressure in the respiratory system, which is used to trigger the device. Depending on the condition of the patient, a trigger pressure between −1 and −5 cmH_2_O is configured for this mode of operation. As a safety feature, if the inhale attempt is not detected within a fixed period (set by the respiration rate and the *I*:*E* ratio), the device automatically defaults to the CM mode and notifies the operator via an alarm. To test this mode, we used 500 mL for the *VT*, a respiration rate of 16 b min^−1^ with an *I*:*E* ratio of 1:2, and a trigger pressure of −1 cmH_2_O. To simulate the negative pressure generated in the attempt for inspiration by the lungs, we applied slight positive pressure to the open end of the differential pressure sensor (Figure 2). As shown in **Figure** **6**, when the pressure drops below −2 cmH_2_O, the device starts the ventilation and delivers the set *VT*. In the third cycle of Figure 6, we made no “attempt” for inhale, and the device defaulted to the controlled mode and operated continuously as expected (Figure 6).

Our design rationale is based on optimizing the performance (e.g., accuracy, portability, and cycle life), simplicity, time to manufacture, scalability, and cost while utilizing generic and readily available components. Unlike pneumatically powered ventilators (e.g., Bird Mark 7, Bio‐Med MVP‐10, and Servo‐I), the driving force in the IPAM ventilator is provided by a robust high precision stepper motor, which makes it portable and reliable for prolonged use. To simplify the manufacturing procedure, we avoided 3D printing any of the components and used readily available materials (Figure 2). We achieved a total bill of materials (BOMs) cost of less than $400 USD for making the prototype (materials and methods).

The IPAM ventilator does not introduce any new modes of operation for ventilators. In other words, standard parameters and modes that are available on any ventilator are used in our solution. Therefore, the adoption of this device could be almost instantaneous. We anticipate most medically trained operators should be able to operate this without any further training or instructions. In the current case of public health emergency, governmental health organizations have relaxed the approval process for mechanical ventilators. For example, Health Canada stated that “a summary of clinical evidence, including clinical study results and/or literature review, in accordance with section 4(1)(g) (i.e., the known information in relation to the quality, safety, and effectiveness of the device) should be provided if and only if the ventilator includes novel features and concepts. Otherwise, the ventilator is an established technology, and clinical study data are not required.”^[^ ^16^, ^17^ ^]^

In designing this device, we took into consideration the safety guidelines for designing a ventilator and incorporated multiple safety features in our design. These measures include the following: 1) The pressure is monitored continuously, and if it passes a certain level that is set in the software, the alarm is triggered to notify the operator. For the first level (currently set at 20 cmH_2_O), the alarm signals periodically, and the device continues to function. For the second level (presently set at 40 cmH_2_O), the alarm signals continuously and halts the machine mid‐operation. A mechanical safety valve is used to release the pressure should this situation occur. 2) In the assisted mode, if the device does not detect an attempt for inspiration from the patient, it automatically switches to the controlled mode and operates according to the set values. 3) In the event of an electronic failure, by default, the stepper motor turns off. This feature enables the operator to actuate the glass syringe easily from the crank joint manually. In this case, the *VT* can be read from the measurement markings on the glass syringe. 4) In the event of mechanical failure, the operator can immediately disconnect the device from the breathing circuit and use the already attached BVM to perform the ventilation manually. 5) The *VT* is limited by two limit switches. The limit switches change the motor's direction of rotation when toggled. One of them is used to recalibrate the position of the motor in every breathing cycle. Moreover, the limit switches prevent the motor from going past a certain angle, which could potentially damage the device. 6) The disassembly process of the device for maintenance and cleaning is very straightforward. Moreover, glass is relatively chemically non‐reactive and resistant to disinfectants.

Historically, to make a ventilator, there is a remarkably high barrier of entry due to its targeted field being very niche and specific (i.e., medical field). The high level of regulations and requirements to make and sell such medical devices adds further to this entry barrier. We are achieving a potential advantage by substantially lowering the barrier of entry to manufacturing, deploying, and owning a mechanical ventilator. The COVID‐19 pandemic has brought to light the need for simpler and readily available devices that can address the urgency and impact of the situation rather than only relying on high‐end all‐rounded “perfect” devices.

We focused our fundamental design on addressing the demand for ventilators in life‐death situations. Therefore, only the core and essential features, such as the safety features mentioned, and the two modes of operation are implemented. Contributing to the cost of high‐end ventilators is the additional quality of life improvements and features that come with the device. Common examples include capnograph, blood oximeter, additional storage for data logging/analysis, larger and more sophisticated LCD interface, ingress protection rating, and impact protection resistance rating. We avoided using custom made parts (e.g., 3D printed components, machined gears, and injection‐molded pieces) and utilized off‐the‐shelf components to cut down the cost further. For example, the precision machining of rigid materials such as Teflon or metals for making a 1000 mL plunger/barrel is a subtractive manufacturing method that is time‐consuming, labor‐intensive, and expensive.

The size and weight of the device are close to that of the commercially available portable ventilators running on pressurized gas tanks (Supporting Information). We believe that it is possible to reduce the size and weight by further engineering the design and selecting lighter but durable materials.

The demand for this device in underdeveloped countries will be much higher due to the sheer cost of high‐end first‐class ventilators. Moreover, underdeveloped countries typically have a higher population density, which, in turn, creates a higher demand for the number of medical devices. In emergencies such as pandemics where the need for ventilators overwhelms the number of ventilators available, our device can act as a temporary or substitute to high‐end medical grade ventilators. Moreover, these ventilators could be part of the first aid or medical kit in large companies or institutions, such as universities and airports. Another possible application would be in education and training institutions where hands‐on experience and time with such devices are not only required but beneficial. Considering the precision for the *VT* delivery that we achieved, we anticipate that our proposed ventilator can be used as an anesthesia ventilator as well.

## 2 Conclusion

In this work, we presented a low‐cost, high performance, and straightforward mechanical ventilator that can be deployed in public health emergencies rapidly. We successfully mimicked the functionality of the two primary modes (i.e., CM and AM) of operation for modern ventilators. In addition, we achieved the accuracy and consistency of a certified commercialized ventilator. To further improve on the device, more modes of operation can be implemented easily as the underlying design and architecture accommodate these alternative modes. In addition, sensors for monitoring the blood oxygen saturation level, exhale CO_2_ level, temperature, electro‐cardiogram, and heartbeat can be easily integrated.

## 3 Experimental Section

3.1

3.1.1

##### Hardware Architecture

The artificial respirator device is constructed of eight major components. The specifications and rationale behind choosing these components are explained in the following.

*Stepper motor:* The maximum safe pressure that can be exerted on the respiratory system is 40 cmH_2_O. To generate this pressure with the glass syringe (plunger diameter of 82 mm), we need a force of 20.7 N. To deliver 1000 mL air, we need an arm length of 95 mm, which gives a torque requirement of 1.97 N m for the motor. Considering that the pressure transmission is not always 100%, we chose a Nema 23, bipolar, 3 N m stepper motor. Stepper motors with higher torque ratings can be utilized for lower respiration rates and *I:E* ratios.

*Motor driver:* We chose a digital stepper driver with a peak current rating of 4.2 A to drive the stepper motor with our microcontroller. The inputs of the driver are optically isolated from the microcontroller to prevent backpropagation of back electromotive force noise or any electromagnetic (EM) noise from the stepper motor. We used 400 pulse rev^−1^ setting to achieve a balance between smooth motion and stressing the microcontroller.

*Crank‐shaft linkage:* We made the crank‐shaft linkage from the components shown in Figure 1. The crank‐shaft linkage components include a clevis rod end, a piece of high‐strength steel threaded rod, a partially threaded rod end bolt, a ball joint linkage, a medium‐strength steel hex nut, a high‐strength steel threaded rod, a piece aluminum sheet, and a flange mount shaft collar to connect the crank to the stepper motor.

**Figure 7:**
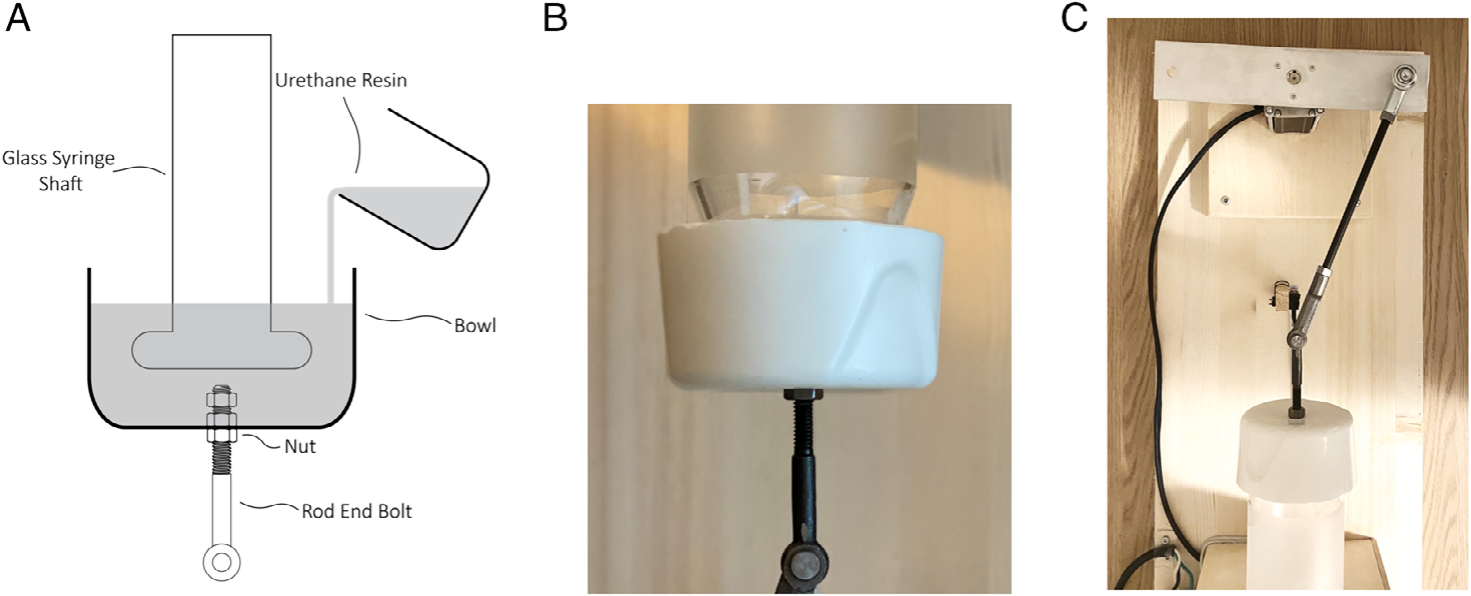
The procedure for attaching the rod end bolt to the top of the glass syringe plunger. A) After the precursors of the urethane resin are mixed and degassed in a desiccator, it is poured to the bowl, which holds the rod end bolt. Two nuts are used inside the container to make it easy to unscrew the rod end bolt if needed. Items are not to scale on the drawing. B) The result of the procedure in (A) showing the attachment. The diameter of the plunger is 82 mm. C) Top view of the crank‐shaft linkage connecting the stepper motor to the plunger of the glass syringe.

To join the top end of the glass syringe to the rod end bolt, we used a fast curing urethane resin with similar performance and mechanical characteristics to acrylonitrile butadiene styrene plastic. **Figure** **7** shows the procedure.

*Glass syringe:* We used a 1000 mL glass syringe manufactured by BLG. The syringe has a 15 mm opening at the tip, which is large enough not to slow down the system for fast actuation rates. Any low friction material or design for the plunger/barrel setup can be a good alternative.

*Power supply:* A 48 V switching power supply with a current rating of 10 A is used to power the stepper motor.

*Microcontroller:* We used an 84 MHz, 32 bit Advanced RISC Machine core microcontroller.

*Sensors:* A differential pressure sensor (Honeywell HSCDRRN160MDSA3) is used to monitor the pressure via the serial peripheral interface.

*Interface:* A 2.4 in. thin‐film transistor color display was used for this work with 12 pushbutton switches and two limit switches. The pushbutton switches are soldered on a printed circuit board with no debounce capacitors. The limit switches are used to enable homing and recalibration of the stepper motor's position in each breathing cycle. Shield wires are used to eliminating EM interference of the stepper motor with the limit switch signals. For the alarm, we used an internally driven piezo buzzer with an operating frequency of 4.1 kHz (C8 on Piano).

*Setup design:* We used pine lumber (3/4″, 12″ × 36″) for the chassis of the device and pine slabs and studs for mounting the glass syringe, limit switches, and making the housing for the electronics. For the final commercialized device, these components will be hosted in an enclosure.

**Figure 8:**
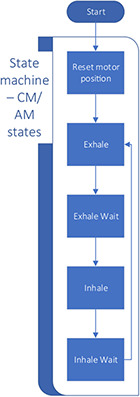
Software state diagram and flow.

*Software architecture:* We designed and implemented two similar state machines for the CM and AM. The following section describes the high‐level logic of the state machines. **Figure** **8** shows the high‐level logic of the CM and AM states. Both modes have the same architecture but with slightly different logic. Details of each state are described as follows.

*Reset motor state:* This state is only executed once after the transition from the “Idle” state to the “CM/AM” states. The goal of this state is to calculate all cycle parameters and move the motor clockwise (CW) to a known 180° position. This position is the same position in which the motor will be in at the end of the inhale cycle. The cycle parameters are calculated in this state. This includes the motor's speed, final rotation steps, and breathing cycle wait times.

*Exhale state:* This state starts the exhale cycle and moves the motor counterclockwise (CCW). This state ends once the stepper motor reaches the final rotation steps.

*Exhale wait state:* This state holds the motor in position for a set period. This period is determined at the start of the cycle from the *I*:*E* ratio, respiration rate, and the *VT* parameters. In the AM, the exhale wait state has additional logic to monitor pressure to detect the patient's attempt to inhale.

*Inhale state:* Similar to the reset state, the motor is moved CW back to the 180° position in this state. The start of the inhale state is also considered the beginning of a new breathing cycle. Therefore, any setting parameters such as respiration rate, *I*:*E* ratio, and *VT* will be updated.

*Inhale wait state:* Similar to the Exhale Wait state, the motor is held in position for a set period of time. This state also calculates and displays the maximum flow rate, peak pressure, PEEP, and plateau pressure.

The breathing cycle is then repeated until the device is stopped, rebooted, or powered off.

*Cost analysis:* The list of the materials and the cost to build a prototype of the device is tabulated in **Table** **2**. The cost amount per component displayed in this table is the cost for 1 unit of each component. The total BOM can be significantly reduced if purchased and assembled in larger quantities. Based on estimated quotes we received from the suppliers, if the components are purchased in units of 1000, the total BOM per device drops to approximately $250/device.

**Table 2:** BOM for the ventilator

Components	Numbers used	Unit price [USD]
Power supply	1	$14.27
Stepper motor	1	$33.75
Motor driver	1	$23.64
1000 mL glass syringe	1	$200.50
Microcontroller	1	$15.99
LCD	1	$2.50
Pushbuttons	12	$1.79
Limit switches	2	$4.97
Buzzer	1	$6.09
Rod end bolt	1	$6.32
Clevis rod ends	1	$5.15
Ball joint linkage	1	$5.21
1 ft high‐strength steel threaded rod	0.5	$9.33
Pressure sensor	1	$35.70
Shaft collar	1	$2.00
Miscellaneous (wood, bolts, screws, etc.)	–	$20.00
Total		$387.21

## Conflict of Interest

The authors declare no conflict of interest.

## Author Contributions

S.M.M. conceived the design, built the hardware, designed the software algorithm, and performed all the experiments. D.S. implemented the software. S.M.M. and D.S. performed the data analysis. R.L. Provided guidance. All authors contributed to the writing of the work.

## Supporting information

Supplementary Material

Supplementary Material

Supplementary Material

Supplementary Material

Supplementary Material
