# Supplementary material for: Inverse Pneumatic Artificial Muscles for Application in Low‐Cost Ventilators
Source: Adv Intell Syst. 2020 Oct 30;3(1):2000200. doi: 10.1002/aisy.202000200 (PMC7645954; doi:10.1002/aisy.202000200)
Supplement: Supplementary file 5 — Supplementary Material [file AISY-3-0-s005.docx]

Copyright WILEY-VCH Verlag GmbH & Co. KGaA, 69469 Weinheim, Germany, 2018.

*Supporting information for*

**Inverse Pneumatic Artificial Muscles for Application in Low-Cost Ventilators**

Seyed M Mirvakili^1,*^, Douglas Sim^2^, Robert Langer^1,3,4,5,*^

^1^ Koch Institute, Massachusetts Institute of Technology, Cambridge, MA 02139, USA.

^2^ Electrical and Computer Engineering Department, University of British Columbia, Vancouver, BC, Canada.

^3^ Department of Chemical Engineering, Massachusetts Institute of Technology, Cambridge, MA, USA.

^4^ Division of Health Science and Technology, Massachusetts Institute of Technology, Cambridge, MA, USA.

^5^ Institute for Medical Engineering and Science, Massachusetts Institute of Technology, Cambridge, MA, USA.

* Corresponding authors. Emails: seyed@mit.edu, sm.mirvakili@gmail.com, rlanger@mit.edu

**The PDF file includes:**

Data Summary for Test Cases

Dynamics of the Respiratory System

Actuator Kinematics

Size and Mass Specifications

Table S1. Result summary for different setting configurations.

Fig. S1. Diagram of the geometry of the crank-shaft linkage.

Fig. S2. Tidal volume as a function of body height for male and female patients.

**Other Supplementary Material for this manuscript includes the following:**

Movie S1 (.mp4 format). Controlled Mode of operation 1

Movie S2 (.mp4 format). Controlled Mode of operation 2

Movie S3 (.mp4 format). Assisted Mode of operation

Movie S4 (.mp4 format). Failure Management

**Data Summary for Test Cases**

We performed 13 experiments with the test lung to evaluate the performance of the IPAM Ventilator for various scenarios. The results are plotted in Figure 4. The values for the set parameter and measured data for each cycle are tabulated in Table S1.

Table S1 – Result summary for different setting configurations. Each experiment is performed for ten consecutive cycles.

| Test No. | Set Parameters | | | | | Measured Values | | |
| --- | --- | --- | --- | --- | --- | --- | --- | --- |
|  | *VT* (mL) | *RR* (b/min) | *I*:*E* ratio | *End-inspiratory hold* (%) | *PEEP* (cmH_2_O) | *P_peak_* (cmH_2_O) | *P_plateau_* (cmH2O) | *F_max_* (L/min) |
| 1 | 750 | 10 | 1:2 | 50 | 5.22± 0.23 | 41.53±0.44 | 37.69± 0.64 | 58.61± 0.02 |
| 2 | 550 | 18 | 1:2 | 50 | 5.64±0.13 | 33.24± 0.66 | 22.98±0.54 | 82.00±0.08 |
| 3 | 400 | 24 | 1:2 | 50 | 5.92±0.07 | 28.00±0.41 | 21.11±0.06 | 85.46 |
| 4 | 300 | 30 | 1:2 | 50 | 6.04±0.04 | 25.16±1.61 | 19.01±0.25 | 81.92±0.14 |
| 5 | 550 | 10 | 1:2 | 50 | 5.45±0.15 | 24.78±0.23 | 22.01±0.21 | 45.59±0.01 |
| 6 | 550 | 12 | 1:2 | 50 | 5.60±0.13 | 26.31±0.22 | 22.5±0.23 | 54.70±0.02 |
| 7 | 550 | 14 | 1:2 | 50 | 5.63±0.12 | 28.14±0.42 | 22.92±0.36 | 63.82±0.02 |
| 8 | 550 | 16 | 1:2 | 50 | 5.69±0.11 | 30.64±0.61 | 23.18±0.50 | 72.92±0.04 |
| 9 | 500 | 13 | 1:1 | 50 | 5.75±0.08 | 23.11±0.27 | 20.70±0.03 | 36.58±0.01 |
| 10 | 500 | 13 | 1:2 | 50 | 5.61±0.08 | 25.20±0.34 | 20.95±0.07 | 54.83±0.01 |
| 11 | 500 | 13 | 1:3 | 50 | 5.54±0.16 | 28.59±0.40 | 21.10±0.11 | 73.06±0.05 |
| 12 | 500 | 13 | 1:4 | 50 | 5.30±0.13 | 33.87±0.27 | 21.10±0.52 | 91.37±0.05 |
| 13 | 500 | 13 | 1:5 | 50 | 5.20±0.13 | 39.52±1.11 | 20.95±0.91 | 109.45±0.10 |

For experiments 1 to 4, we kept the *I:E* ratio constant at 1:2 but increased the *RR* from 12 b/min to 30 b/min while decreasing the *VT* from 750 mL to 300 mL. As we lowered the tidal volume, both the peak pressure and plateau pressure dropped as well. This correlation can be explained by the fact that less air is moved to the test lung; therefore, a smaller pressure is developed inside it. For experiments 5 to 8, we kept the tidal volume and *I:E* ratio constant and increased the respiration rate. As expected, the flow rate increases as well as the difference between the peak pressure and plateau pressure increases. To show this effect in more depth, for experiments 9 to 13, we kept the *VT* at 500 mL and *RR* at 13 b/min but increased the *I:E* ratio from 1:1 to 1:5. Similar to the previous case, since the volume is kept constant, the plateau pressure is also constant; however, the peak pressure increases, which can be explained by the fact the resistive pressure is increasing due to the increase in flow rate. More details are provided in the next section. This behavior of the test lung is similar to that of the human lungs.

**Dynamics of the Respiratory System**

The lung is a porous structure made of soft tissues and a complex network of airway branches. Due to the inhomogeneity of its structure, the pressure developed in the lungs has three components:

- *Resistive Pressure (P_resistive_)* which is the contribution from the resistive properties of the respiratory system. Viscous and turbulent losses related to the flow of gas through the airway tree and the deformation of parenchymal and chest wall tissues are probably the main contributors to the resistive pressure (*1*). At small flow rates, the resistive pressure is a linear function of the flow rate (*V̇*). However, at higher flow rates (*e.g.*, low *I*:*E* ratios, exercising), the resistive pressure scales nonlinearly, often in the form of *k_1_V̇* + *k_2_V̇^2^*, where *k_1_* and *k_2_* are determined empirically.
- *Elastic Pressure (P_elastic_)* which is the contribution from the recoil of the lungs and chest wall to their relaxed states when inflated with air either by contraction of the diaphragm and intercostal muscles or by mechanical insufflation (*e.g.*, a ventilator). Therefore, the elastance of the respiratory system (*E_rs_*) can be seen as a linear summation of the elastance from the chest walls (*E_cw_*) and the elastance from the lungs (*E_l_*). The reciprocal of the respiratory system’s elastance gives its compliance (*C_rs_*). The elastic pressure is a function of the tidal volume.
- *Inertial Pressure (P_inertial_)* which is the contribution from the inertial forces from the chest wall, airway tree, and parenchymal tissues. The inertial pressure is a function of volume’s acceleration (*V̈*).

Therefore, the total pressure is (*2*, *3*):

*P* = *IV̈* + *RV̇* + *EV* + *P_o_*, (1)

where *I*, *R*, and *E* are the inertial, elastic, and resistive properties of the respiratory system. The *P_o_* is the distending pressure at the end of expiration.

The resistance (*R*) can be estimated from the difference between the peak pressure and plateau pressure divided by the flow rate. Any alterations in *P_resistive_* (for a specified flow rate) can reflect changes in the airway caliber (*4*).

At zero flow rate, the elastance of the respiratory system can be determined from the difference between the plateau pressure and the *PEEP* divided by the tidal volume. It is important to note that the dynamic elastance is higher than static elastance in general, which is due to the viscoelasticity and gas redistribution. Figure S1 illustrates the contribution of each component of the pressure in the respiratory system.


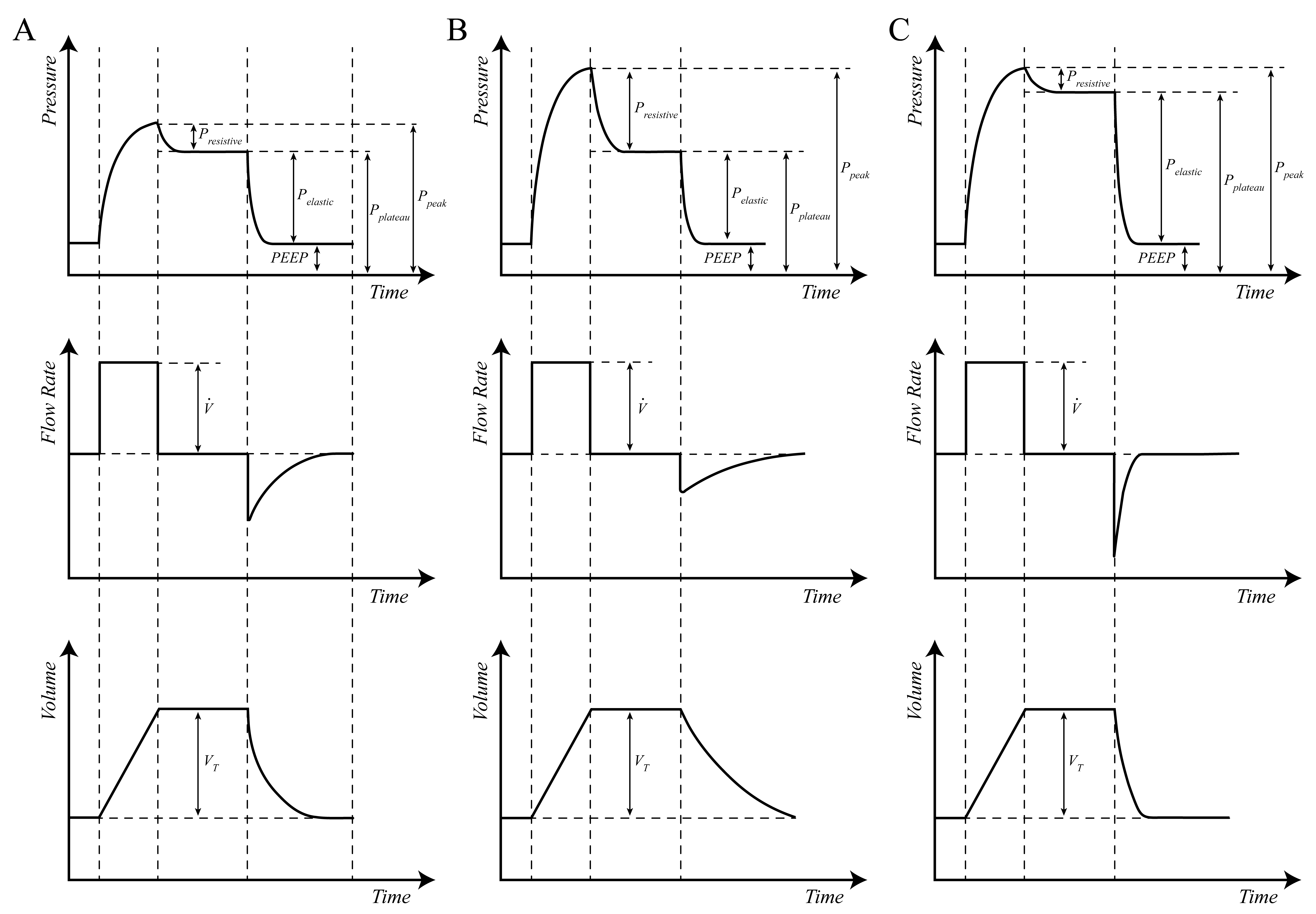


Figure S1 – Pressure, flow rate, and volume profiles in volume-controlled ventilation (constant flow rate). (A) Pressure, flow rate, and volume profiles for a healthy patient. In this the case, the resistive pressure has a small contribution to the peak pressure. While the elastic pressure has the largest contribution. (B) The peak pressure is increased while the plateau remains similar to that of in (A). This indicates an increase in the resistive pressure. This condition occurs in cases such as bronchospasm, mucous plug, retained secretions, and ETT tip occlusion. (C) The elastic pressure is increased in this case, while the resistive pressure contribution is similar to that of in part (A). Conditions such as ARDS, pneumonia, pneumothorax, and pulmonary oedema lead to an increase in the elastic pressure.

The predicted body weight (PBW) for male and female patients can be estimated from the height of the patients according to the ideal body weight equations described in the main text. Figure S2 illustrates the tidal volume as a function of the patient’s height for different gravimetric tidal volumes. As shown in Figure S2, the range for tidal volume is between 70 mL to 840 mL.

Figure S2 – Tidal volume as a function of body height for different gravimetric tidal volumes for male and female patients.

**Actuator Kinematics**


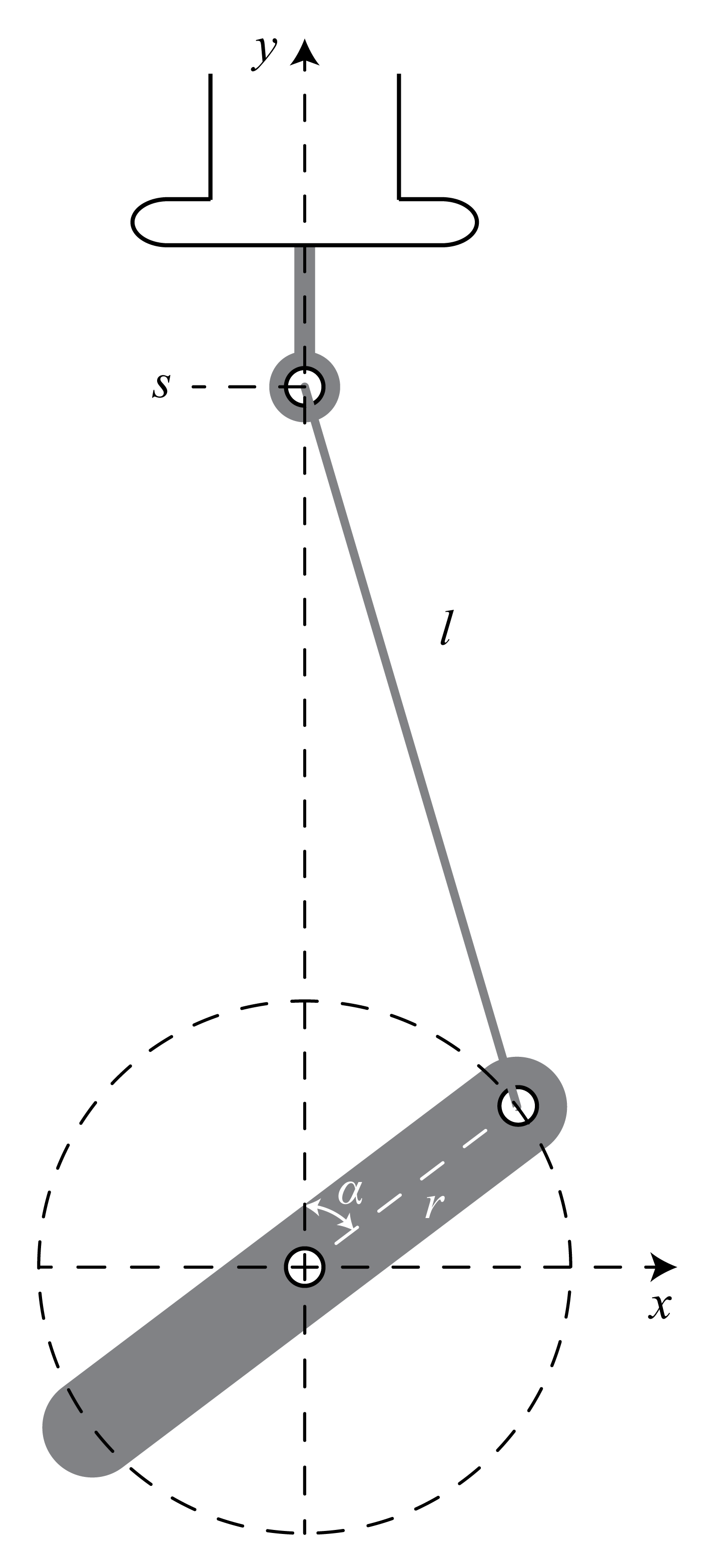
 The position of the plunger is almost proportional to the cosine of the angle between the crank axis and axis of the plunger (Figure S3). From the cosine law, we can find the position of the plunger (*s*) as a function of the crank radius (*r*) – which is half of the linear stroke, and length of the connecting rod (*l*) as the following equation suggests:


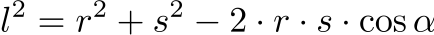
 (2)

The position of the plunger corresponds to the volume that the syringe can deliver. By rearranging the terms in equation 2, we find the *s* to be:


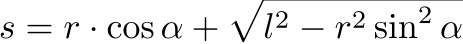
 (3)

Figure S3 – Diagram of the geometry of the crank-shaft linkage.

Therefore, the tidal volume (*VT*) as a function of rotation stroke (*α_i_ - α_f_*) can be found to be:


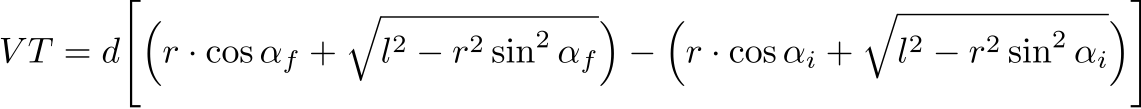


(3)

where *d* is the volume capacity of the glass syringe (*i.e.*, 1000 mL) over the length of the plunger that should travel inside the barrel to achieve that volume capacity (*e.g.*, the amplitude of the maximum linear stroke). For extreme cases of *α* = 0° and *α* = 180° we obtain *VT* = 2*rd*. Since *r* is half of the maximum linear stroke, we obtain *VT* = 1000 mL as expected.

**Size and Mass Specifications**

The weight of our device is 10.4 kg, and its dimension is *w* = 300 mm, *l* = 915 mm, *h* = 250 mm, which gives a volume of 0.0686 m^3^ or 68.6 L. The dimensions for commercialized ventilators are listed below:

Table 2 - Size and mass specifications of the device in this work and commercially available devices

| Device | Width (mm) | Length (mm) | Height (mm) | Volume (L) | Weight (kg) | Gas tank^*^ (L) | Gas tank^*^ (kg) |
| --- | --- | --- | --- | --- | --- | --- | --- |
| Maquet Servo-I | 205 | 365 | 733 | 54.8 | 20 | 6.5 | 3.5 |
| Hamilton C6 | 360 | 250 | 809 | 72.8 | 18.3 | 6.5 | 3.5 |
| Puritan Bennett™ 560 | 235 | 315 | 154 | 11.4 | 4.5 | 6.5 | 3.5 |
| This work | 300 | 915 | 250 | 68.6 | 10.4 | Not Needed | Not Needed |

^*^ Physical volume of the gas tank. It can provide a flow rate for ventilation between 2 to 22 hours – depending on the flow rate.

In most hospitals, the pressure source is from the pressure lines, which reduces the portability. Pressurized gas tanks are used to make the devices portable; however, they are often large, heavy and require peripherals such as regulators, adding to the size and weight. The IPAM Ventilator does not need an external pressure source; thus, it can run continuously without disruption. We believe it is possible to reduce the size and mass of the device by replacing wood with lighter and stronger composite materials.

**References:**

1. D. W. Kaczka, E. P. Ingenito, B. Suki, K. R. Lutchen, Partitioning airway and lung tissue resistances in humans: effects of bronchoconstriction. *J. Appl. Physiol.* **82**, 1531–1541 (1997).

2. J. H. T. Bates, *Lung Mechanics: An Inverse Modeling Approach* (Cambridge University Press, 2009).

3. D. W. Kaczka, A. A. Colletti, M. H. Tawhai, B. A. Simon, in *Image-Based Computational Modeling of the Human Circulatory and Pulmonary Systems: Methods and Applications*, K. B. Chandran, H. S. Udaykumar, J. M. Reinhardt, Eds. (Springer US, Boston, MA, 2011; https://doi.org/10.1007/978-1-4419-7350-4_10), pp. 375–402.

4. T. J. Pedley, R. C. Schroter, M. F. Sudlow, The prediction of pressure drop and variation of resistance within the human bronchial airways. *Respir. Physiol.* **9**, 387–405 (1970).
